# Near-complete pathologic resolution of untreated papillary thyroid cancer after pembrolizumab-induced thyroiditis

**DOI:** 10.1210/jcemcr/luag030

**Published:** 2026-03-17

**Authors:** Margaret Locke, Sara Sternbach, Nagashree Seetharamu, Charit Taneja

**Affiliations:** Department of Hematology/Oncology, Donald and Barbara Zucker School of Medicine at Hofstra/Northwell, New Hyde Park, NY 11042, USA; Department of Pathology, Donald and Barbara Zucker School of Medicine at Hofstra/Northwell, New Hyde Park, NY 11042, USA; Department of Hematology/Oncology, Donald and Barbara Zucker School of Medicine at Hofstra/Northwell, New Hyde Park, NY 11042, USA; Department of Endocrinology, Donald and Barbara Zucker School of Medicine at Hofstra/Northwell, New Hyde Park, NY 11042, USA

**Keywords:** papillary thyroid cancer, immune checkpoint inhibitors, pembrolizumab

## Abstract

Immune checkpoint inhibitors (ICIs) have revolutionized treatment for multiple cancers. However, research into the therapeutic potential of ICIs for differentiated thyroid cancer (DTC) is sparse. We present a case of a 55-year-old woman with untreated papillary thyroid cancer who received pembrolizumab as part of treatment for her breast cancer and then developed pembrolizumab-induced destructive thyroiditis, leading to near-complete pathologic resolution of her thyroid cancer. This suggests therapeutic potential to use ICIs in patients with DTCs who are not candidates for standard therapies such as surgical resection, and as an additional agent for patients with advanced metastatic DTC.

## Introduction

Initial management for differentiated thyroid cancer (DTC) can usually vary from active surveillance to hemithyroidectomy or total thyroidectomy, with selective use of adjuvant radioactive iodine (RAI) for patients at intermediate and high risk of recurrence [1]. For patients who are unable to undergo initial surgical resection or with advanced malignancy refractory to RAI, alternative treatment options are limited. Select patients with advanced, metastatic RAI-refractory thyroid cancer are candidates for treatment with tyrosine kinase inhibitors.

One area of active but limited investigation is the role of immune checkpoint inhibitors (ICIs) in this space. Although ICIs are now increasingly used across multiple tumor types, their role in management of DTC is not well defined. Currently, pembrolizumab is approved by the US Food and Drug Administration for use in unresectable or metastatic solid tumors with tumor mutational burden (TMB) greater than 10 mutations/megabase and after consideration first for tyrosine kinase inhibitors and other targeted agents [2]. Case reports describing patients' responses to checkpoint inhibitors are thus valuable in the optimizing selection of patients who may benefit from ICIs, especially in cancer types for which these therapies are not frequently used. Recent studies have shown some utility of ICIs in patients with anaplastic thyroid cancers, but data in patients with DTC remain sparse [3, 4]. Here we present a case of destructive thyroiditis from pembrolizumab used for breast cancer leading to near-complete pathologic resolution of the patient's untreated papillary thyroid cancer (PTC).

## Case presentation

We present the case of a 55-year-old woman with stage II triple-negative breast cancer diagnosed after a screening mammogram showed a 12-mm noncalcified mass in the left breast. Ultrasound-guided biopsy revealed poorly differentiated invasive ductal carcinoma (estrogen receptor negative, progesterone receptor negative, human epidermal growth factor receptor 2 negative, Ki-67 index 91%). On initial staging computed tomography scans, she was noted to have an incidental right thyroid nodule. She did not have a visible or palpable thyroid swelling. She denied dysphagia, dyspnea, or hoarseness of voice. She denied any family history of thyroid cancer or history of radiation exposure to her neck.

## Diagnostic assessment

**Figure 1:**
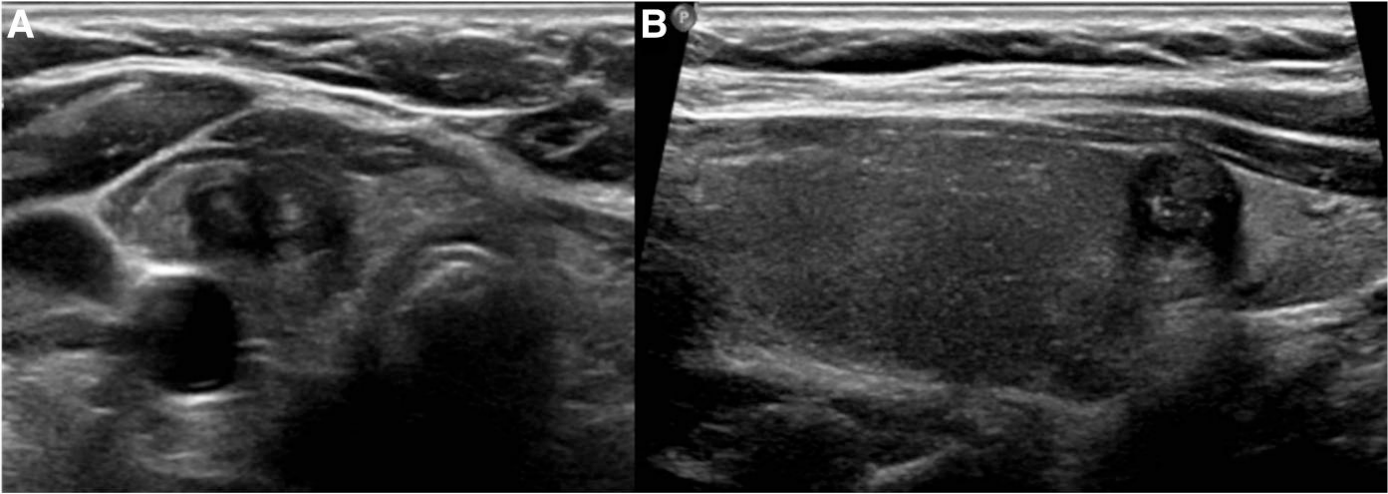
Thyroid ultrasound: A, transverse and B, sagittal views showing right lower pole 1.4 cm markedly hypoechoic nodule with irregular/lobulated margins and punctate echogenic foci (American College of Radiology Thyroid Imaging and Data System 5, high suspicion).

Thyroid ultrasound demonstrated a right lower pole, 1.0 × 0.8 × 1.4-cm markedly hypoechoic nodule with irregular/lobulated margins and punctate echogenic foci, classified as an American College of Radiology Thyroid Imaging and Data System (ACR TI-RADS) 5 nodule (Fig 1). There was no abnormal cervical lymphadenopathy identified on neck ultrasound. She had a normal thyrotropin (TSH) of 1.60 µU/mL (normal range, 0.27-4.20 µU/mL). Ultrasound-guided fine-needle aspiration revealed a cellular aspirate showing monolayer sheets and clusters of neoplastic cells with oval enlarged and irregular nuclei, showing nuclear grooves, powdery chromatin, rare intranuclear inclusions, and micronucleoli consistent with Bethesda category VI cytology favoring PTC.

## Treatment

**Figure 2:**
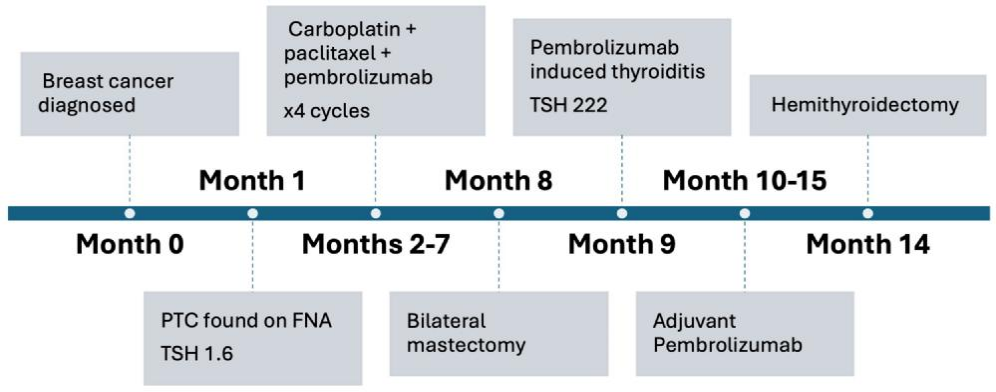
Timeline of events from breast cancer diagnosis to thyroid surgery.

For treatment of stage II triple negative breast cancer per KEYNOTE-522 regimen, the patient received 4 cycles of neoadjuvant carboplatin + paclitaxel + pembrolizumab. Pembrolizumab was dosed at 200 mg intravenously every 3 weeks. This was followed by mastectomy and adjuvant pembrolizumab, which achieved a pathologic complete response. The timeline of events is shown in Fig 2.

**Figure 3:**
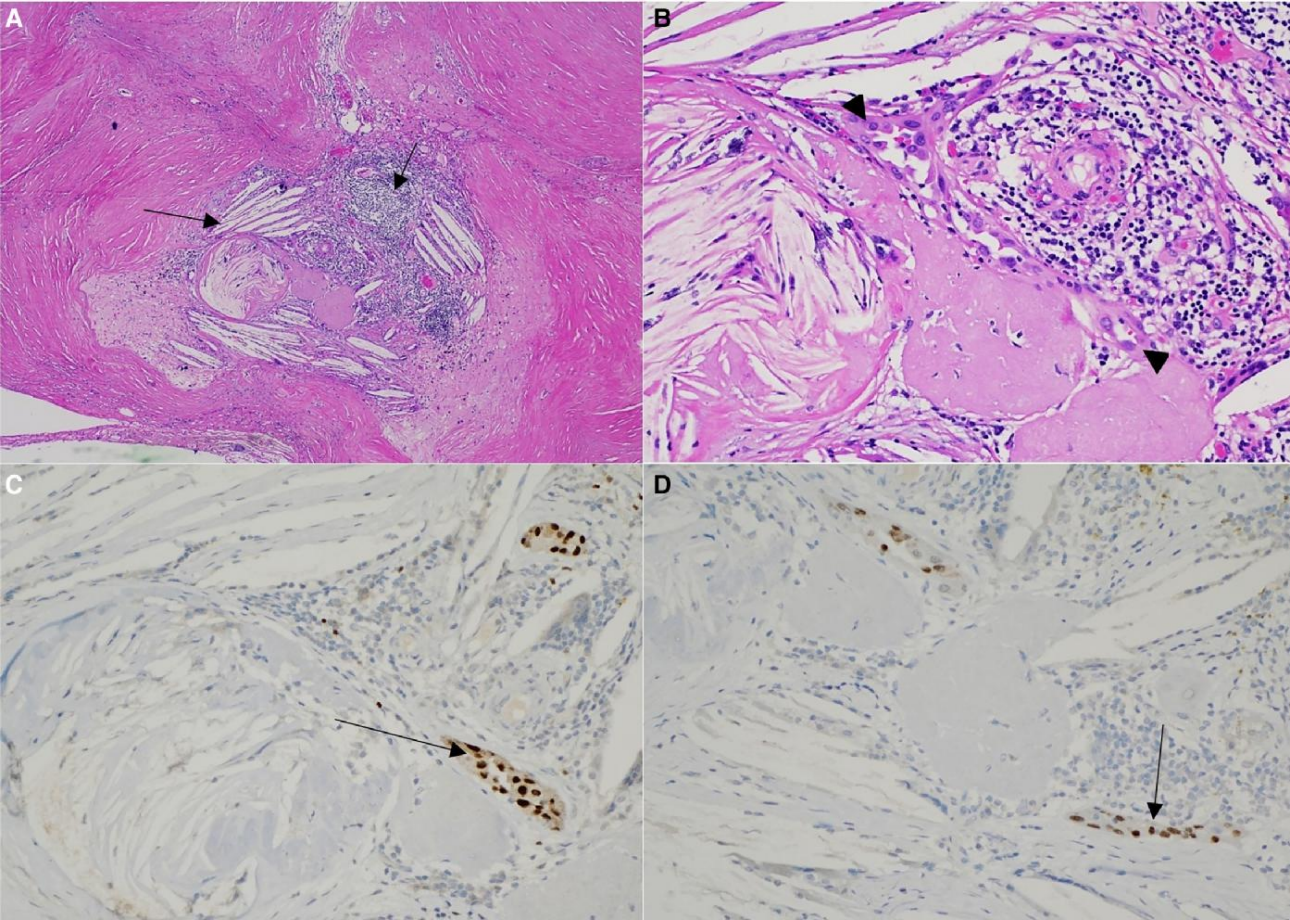
Focus of tumor cells with surrounding fibrosis, cholesterol clefts, and chronic inflammation under A, low power and B, high power. Immunohistochemical staining in tumor cells is positive for C, PAX-8 and D, TTF-1.

To prioritize breast cancer therapy, immediate surgical intervention for her PTC was deferred and instead active surveillance was initiated, with plans for repeat thyroid sonography every 6 months. Thyroid ultrasound approximately 7 months later showed diffuse thyroid atrophy and unchanged size of her biopsy-proven PTC. Around the same time, she developed pembrolizumab-induced thyroiditis with severe hypothyroidism on her monthly laboratory values. Laboratory studies showed TSH 222 µU/mL up from a baseline of 2 µU/mL, free thyroxine less than 0.1 ng/dL (SI: <1.3 pmol/L) (normal range, 0.9-1.8 ng/dL [SI:11-23 pmol/L]), thyroglobulin less than 0.20 ng/mL (normal range, 0.90-77.30 ng/mL), and thyroglobulin antibodies less than 15.0 IU/mL (normal range ≤115 IU/mL). Cortisol and adrenocorticotropin were within normal limits. She was started on levothyroxine 150 mcg daily (∼1.6 mcg per kg body weight) with subsequent normalization of thyroid function tests on medication. After a total of 12 months on pembrolizumab, she opted to undergo right hemithyroidectomy for definitive management of PTC as thyroid sonography 5 months prior to surgery showed unchanged nodule size. Pathology showed extensive fibrosis with degenerative changes and cholesterol clefts, and only scant residual tumor cells measuring approximately 0.1 cm. Immunohistochemical staining showed the scant tumor cells positive for PAX-8 and TTF-1, but negative for thyroglobulin and GATA-3 (Fig 3).

## Outcome and follow-up

The patient had an uneventful recovery after hemithyroidectomy and remained on thyroid hormone supplementation for hypothyroidism. Thyroglobulin and thyroglobulin antibody levels remain undetectable after hemithyroidectomy likely secondary to underlying destructive thyroiditis process. Neck ultrasound around 6 months after thyroid surgery showed no evidence of structural disease in the right thyroid bed, and an atrophic left lobe consistent with prior thyroiditis. She remains in remission from breast cancer, not currently on any oncologic therapies.

## Discussion

Although initial treatment of DTC usually entails surgery with or without RAI ablation, options are limited for patients with advanced, unresectable disease or for those who are poor surgical candidates due to additional comorbidities. The somewhat serendipitous near-resolution of this patient's previously untreated PTC after treatment with pembrolizumab as part of her breast cancer therapy, however, suggests possible therapeutic potential of ICIs in this space by inducing destructive thyroiditis.

The role of ICIs in treatment for DTC has been investigated in only a few small studies, and not in the upfront setting. Historically, DTC is poorly responsive to chemotherapy and we do not believe that the therapeutic effect from thyroiditis was related to the patient’s prior chemotherapy, which had finished 2 months earlier. There have been case reports similar to ours reporting a therapeutic effect on PTC from ICIs used for another indication (eg, colon adenocarcinoma, renal cell carcinoma, hepatocellular carcinoma) resulting in size reduction of unresected PTC related to immune-mediated thyroiditis [5-7]. To our knowledge, ours is the first such report with histopathologic confirmation of regression of tumor cells and near-complete replacement by fibrosis and degenerative changes likely associated with underlying ICI-induced thyroiditis. On the contrary, there are scattered case reports with suggestions of new-onset PTC after pembrolizumab use and anaplastic transformation after nivolumab use [8, 9]. The literature in this regard is sparse, and further research is needed to delineate the molecular-level changes that ICIs can induce on tumor cells, whether this is therapeutic or deleterious.

The tolerability and efficacy of pembrolizumab in patients with DTC were assessed as part of the phase Ib KEYNOTE-028 trial, which evaluated patients across 20 different tumor types who received pembrolizumab at a dose of 10 mg/kg every 2 weeks [10]. Twenty-two patients enrolled who had advanced thyroid cancer, and treatment-related adverse events (trAEs) were observed in 82% of patients, and only 1 patient experienced a grade 3 or higher trAE (colitis), and no treatment discontinuations as a result of trAEs. Overall response rate (ORR) was only 9% with a progression-free survival (PFS) of 7 months. This demonstrated a manageable safety profile but a somewhat low ORR in only a minority of DTC patients, and at much higher doses than received by the patient in our case. The phase 2 KEYNOTE-158 trial used pembrolizumab at a dose of 200 mg every 3 weeks and evaluated patients across multiple tumor types; this dose was similar to what the patient in our case received for breast cancer treatment [11]. In the subgroup of 103 patients with DTC, ORR was 6.8% but 8.7% in patients with a combined positive score greater than 1, median overall survival of 34.5 months, and PFS of 4.2 months. TrAEs occurred in 69% of patients, with grade 3 and above in 14%. Another phase 2 trial evaluating the efficacy of a combination of nivolumab and ipilimumab showed a similar ORR of 9.4% in patients with RAI-refractory DTC (3/32 partial responders), though an exploratory cohort of anaplastic thyroid cancer patients showed an ORR of 30% [12].

Overall, although fairly well tolerated, the ORR for DTC in response to pembrolizumab from these small studies is somewhat low at less than 10%. This number seems to correspond to reports of rates of thyroiditis and other thyroid-related adverse events in patients who received pembrolizumab and other ICIs [13, 14]. Future studies are needed to assess the selection of advanced DTC patients who may be more likely to respond and benefit from ICIs and to identify patient-specific characteristics that may portend better response.

Our case adds to the literature by reporting histopathologic response in a patient with PTC treated with pembrolizumab for an unrelated malignancy. We hypothesize that a longer duration of continued active surveillance of the small PTC in this case may have eventually shown radiologic resolution of her thyroid malignancy and potentially avoided the need for thyroid surgery. Surgical resection in this case adds valuable insight by providing an objective histopathologic outcome after treatment with ICI, and likely a result of ICI-induced thyroiditis that could have been considered a predictor of the response in this case. There is a need to further explore the potential to use ICIs in patients who cannot receive upfront surgery for DTC as a “neoadjuvant” approach, as has been seen in combination with BRAF inhibition in patients with *BRAF*-mutated anaplastic thyroid cancer [15]. Our case also lends guidance in the sequencing of treatment. In patients with intrathyroidal DTC in which a second primary malignancy is present and ICI is planned, it may be beneficial to pursue an active surveillance strategy for the thyroid cancer since ICI use may very well induce a complete radiologic and pathologic resolution in the DTC, potentially helping avoid the need for thyroid surgery altogether in such select cases. Further research is also needed to better elucidate tumor and molecular characteristics of patients who are more likely to develop checkpoint inhibitor–associated thyroiditis and to harness it as a therapy rather than a toxicity.

## Learning points

- ICIs are increasingly being used for multiple tumor types but studies showing efficacy in DTC are lacking.
- ICI use for unrelated malignancy in a patient with an untreated PTC may induce radiologic and pathologic response on follow-up.
- ICI-induced thyroiditis, although often viewed as an adverse event, can have therapeutic potential in patients with DTC.

## Contributors

All authors made individual contributions to authorship. M.L. contributed to conceptualization, data collection, and literature review. S.S. contributed to diagnosis and management of the patient, procuring pathology slides and images. N.S. contributed to conceptualization and supervision. C.T. contributed to diagnosis and management of the patient, conceptualization, supervision, and literature review. All authors reviewed and approved the final draft.

## Data Availability

Data sharing is not applicable to this article as no datasets were generated or analyzed during the current study.

## Abbreviations

DTC: differentiated thyroid cancer
ICI: immune checkpoint inhibitor
ORR: overall response rate
PFS: progression-free survival
PTC: papillary thyroid cancer
RAI: radioactive iodine
trAEs: treatment-related adverse events
TSH: thyrotropin
